# The Rainbow Arching over the Fluorescent Thienoviologen Mesophases

**DOI:** 10.3390/nano12234284

**Published:** 2022-12-01

**Authors:** Giuseppina Anna Corrente, Giuseppe Di Maio, Massimo La Deda, Odda Ruiz de Ballesteros, Bartolo Gabriele, Lucia Veltri, Finizia Auriemma, Amerigo Beneduci

**Affiliations:** 1 Laboratory of Physical Chemistry, Materials and Processes for Industry, Environment and Cultural Heritage, Department of Chemistry and Chemical Technologies, University of Calabria, Via P. Bucci, Cubo 15D, 87036 Arcavacata di Rende, CS, Italy; 2 Laboratory of Inorganic Molecular Materials, Department of Chemistry and Chemical Technologies, University of Calabria, Via P. Bucci, Cubo 14C, 87036 Arcavacata di Rende, CS, Italy; 3 CNR Nanotec, Institute of Nanotechnology, U.O.S. Cosenza, 87036 Arcavacata di Rende, CS, Italy; 4 Dipartimento di Scienze Chimiche, Università di Napoli Federico II, Complesso Monte Sant’Angelo, Via Cintia, 80126 Napoli, Italy; 5 Laboratory of Industrial and Synthetic Organic Chemistry (LISOC), Department of Chemistry and Chemical Technologies, University of Calabria, Via Pietro Bucci 12/C, 87036 Arcavacata di Rende, CS, Italy

**Keywords:** thienoviologens liquid crystals, self-assembly, supramolecular interactions, bulk fluorescence, thermofluorochromism, anticounterfeiting, single layer thermofluorochromic devices

## Abstract

Thermofluorochromic materials exhibit tunable fluorescence emission on heating or cooling. They are highly desirable for applications ranging from temperature sensing to high-security anti-counterfeiting. Luminescent matrices based on liquid crystals are very promising, particularly those based on liquid crystals with intrinsic fluorescence. However, only a few examples have been reported, suggesting ample margins for development in the field, due to the wide range of fluorophores and supramolecular organizations to be explored. Moreover, thermofluorochromic liquid crystals can be tailored with further functionalities to afford multi-stimuli responsive materials. For the first time, herein we report the thermofluorochromism of thienoviologen liquid crystals, already known to show bulk electrochromism and electrofluorochromism. In particular, we studied their photophysics in the 25 °C–220 °C range and as a function of the length of the N-linear alkyl chains, *m* (9 ≤ *m* ≤ 12 C atoms), and the type of anion, *X* (*X* = OTs^-^, OTf^-^, BF_4_^-^, NTf_2_^-^). Interestingly, by changing the parameters *m*, *X* and T, their fluorescence can be finely tuned in the whole visible spectral range up to the NIR, by switching among different mesophases. Importantly, by fixing the structural parameters *m* and *X*, an interesting thermofluorochromism can be achieved for each thienoviologen in a homologous series, leading to a switch of the emitted light from red to green and from white to blue as a consequence of the temperature-induced variation in the supramolecular interactions in the self-assembled phases.

## 1. Introduction

Smart luminescent materials able to exhibit different colors in response to external stimuli have aroused immense attention in the fields of lighting, sensing, displays, and optoelectronics [1,2,3]. These materials are known as fluorochromic, whose prefix depends on the external stimulus to which they are responsive, i.e., thermo-, electro-, vapo-, solvato-, and mechano- [4,5,6]. Thermofluorochromism (TFC) deals with the reversible modulation of the emission color by temperature [7,8,9]. These materials are gaining a lot of attention due to their potential applications in sensors, optoelectronic devices, and anticounterfeiting market, among others [10,11,12,13,14]. The two main strategies employed to afford TFC materials are the use of intrinsically switchable emitters [15], whose photoluminescence modulation depends on their intrinsic sensitivity to temperature changes or the use of emitters in bulk arrangements or embedded into a matrix for which a thermal stimulus changes the environmental structural parameters, thereby inducing a modulation of the photoluminescence, e.g., liquid crystals, gels, and polymers [16]. The main challenges in this field are the development of multithermofluorochromic materials by which a gradual color modulation could be achieved and of TFC materials capable of working in wide temperature ranges [17]. Moreover, TFC materials that respond to further stimuli other than the thermal one are highly desirable for multi-level anticounterfeiting technology [8,13,14,17,18]. Luminescent matrices based on liquid crystals (LCs) have received a great deal of attention because they can be developed either by incorporating a fluorophore in the LC mesophase [16] or by designing LCs with intrinsic photoluminescence [17,18,19,20,21,22]. The last ones are particularly interesting due to their intrinsic light-emitting ability [18] and unique supramolecular organization [19,20]. Lu et al. reported a novel luminescent liquid crystal, (2Z,2′Z)-2,2′-(1,4-phenylene)bis(3-(4-(dodecyloxy)phenyl)acrylonitrile) (PDPA), which shows green, yellow, and orange colors depending on the self-assembled structure. Its luminescence can be tuned by mechanical shearing and thermal annealing, indicating good capability for piezochromic and thermochromic luminescence switching [21]. More recently, Liao and coworkers, showed the thermochromism of two pyrene liquid crystal derivatives, 1,6-Bis((3,4,5-tris(dodecyloxy)phenyl)-ethynyl)pyrene and 1,6-Bis((3,4,5-tributoxyphenyl)ethynyl)-pyrene, which underwent an apparent emission color change from yellow to green as a function of the liquid crystalline phase. The thermochromic behavior of these materials is mainly due to changes in intermolecular interactions [22]. In an effort to design multiresponsive fluorochromic materials, a promising class is that of ionic liquid crystals (ILCs), because they share properties typical of liquid crystals and some properties of ionic liquids (ILs) [23,24,25,26,27,28]. Indeed, in addition to their exceptional mesophase electrochemical properties (high ion conductivity, high electronic charge mobility, reversible redox chemistry), they can be designed to incorporate conjugated rigid cores with specific redox active and optical properties, among which are electrochromism [28,29,30,31,32,33,34,35,36,37,38,39] and electrofluorochromism [37,40,41].

**Figure 1:**
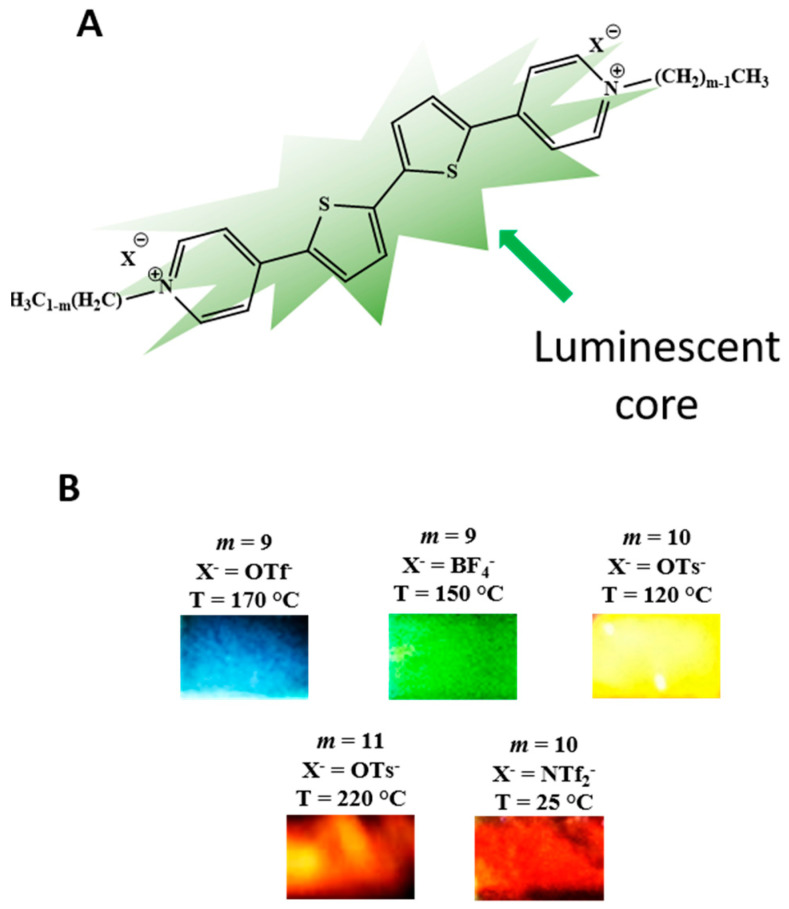
(**A**) Structures of the thienoviologens salts mVX forming ionic liquid crystals, with m indicating the promesogenic alkyl chains attached to the π-extended luminescent thienopyridinium core (V) and X^-^ the anion. (**B**) Photographs of the light emitted (excitation at 430 nm) by thin films formed between two glass substrates by some thienoviologen salts in the LC state, showing the full color palette obtainable as a function of X^-^, m and the temperature (T).

Beneduci et al. [40,41] discovered electrofluorochromism in π-conjugated ILCs by demonstrating that the electrochemical reduction of thienoviologen bistriflimide ILCs, in both columnar and smectic phases, increases fluorescence intensity when the radical cation is formed, while quenching occurs upon further reduction to the neutral species [41]. Furthermore, when monoreduction occurs in the isotropic liquid state, the intensity of the fluorescence increases with a red shift (100 nm) of the light emission due to an electric-field-induced transition to the smectic mesophase. Starting from this knowledge and from the rich thermotropic behavior of the liquid crystalline thienoviologens of the type *m*VX with *m* (9 ≤ *m* ≤ 12 C atoms) defining the length of the promesogenic linear alkyl chains used for pyridine quaternization and X defining the counterion (X = OTs^-^, OTf^-^, NTf_2_^-^, BF_4_^-^) (Figure 1); herein, we studied their thermofluorochromic properties in a wide temperature range (25 °C–220 °C) and as a function of the above structural parameters. Interestingly, by changing the parameters *m*, *X,* and T, their fluorescence can be finely tuned in the whole visible spectral range up to the NIR, by switching among different mesophases. Importantly, by fixing the structural parameters *m* and *X*, an interesting thermofluorochromism can be achieved for each thienoviologen in a homologous series, leading to a switch of the emitted light from red to green and from almost white to blue, as a consequence of the temperature-induced variation of the supramolecular interactions in the self-assembled phases.

## 2. Materials and Methods

### 2.1. Synthesis and Characterization

Thienoviologens 4,4′-(2,2′-bithiophene-5,5′-diyl)bis(1-alkylpyridinium) triflate, tosilate, and triflimide were prepared by metathesis from the corresponding iodide as previously reported [29]. The same procedure was employed for the preparation of 4,4′-(2,2′-bithiophene-5,5′-diyl)bis(1-nonylpyridinium) tetrafluoroborate 9V(BF_4_) starting from 4,4′-(2,2′-bithiophene-5,5′-diyl)bis(1-nonylpyridinium) iodide (for more details see the Supporting information section). The newly synthesized 9V(BF_4_) compound was added to this large series to further evaluate the influence of the anion on the mesomorphic and photophysical properties.

All other materials were commercially available and used without further purification. The compounds were characterized by IR, ^1^H NMR, ^19^F NMR spectroscopy, and HR mass spectrometry. ^1^H NMR spectra were recorded at 25 °C in MeOH-d_4_ on a Avance 300 Bruker NMR spectrometer (Bruker, Milano, Italy) at 300 MHz with Me_4_Si as an internal standard. ^19^F NMR spectra were recorded at 25 °C in MeOH-*d*_4_ on an Avance 500 Bruker NMR spectrometer at 471 MHz with CFCl_3_ as an internal standard. Chemical shifts (δ) and coupling constants (J) are given in ppm and in Hz, respectively. IR spectra were taken with a Shimadzu IR Affinity-1S spectrometer (Shimadzu, Milano, Italy). Mass spectra were obtained using a Q-TOF-MS, equipped with an electrospray ion source (ESI) operated in dual ion mode (Agilent, Milano, Italy). A total of 10 μL of the sample solutions (CH_3_OH) was introduced by continuous infusion at a flow rate of 200 L min^−1^ with the aid of a syringe pump. Experimental conditions were as follows: capillary voltage, 4000 V; nebulizer pressure, 20 psi; flow rate of drying gas, 10 L/min; temperature of sheath gas, 325 °C; flow rate of sheath gas, 10 L/min; skimmer voltage, 60 V; OCT1 RF Vpp, 750 V; fragmentor voltage, 170 V. The spectra data were recorded in the m/z range of 100–1000 Da in a centroid pattern in full-scan MS analysis mode. The MS/MS data of the selected compounds were obtained by regulating diverse collision energies (18–45 eV).

### 2.2. Polarizing Optical Microscopy (POM), Differential Scanning Calorimetry (DSC), and X-ray Powder Diffraction (XRD)

The thermotropic behavior of the sample 9VBF_4_ was studied by POM, DSC, and XRD. Sample textures were observed by a Leitz Laborlux 12 POL polarizing optical microscope (ZEISS, Milano, Italy). Samples were analyzed on thin films formed between cover slides on heating and cooling thermal cycles controlled by a Linkam LTS350 hot stage (Linkam, Salford, UK). DSC thermograms were recorded with a DSC apparatus Mettler DSC-822 (Mettler-Toledo, Milano, Italy), operating in a flowing N_2_ atmosphere at a 10 °C min^−1^ scanning rate. DSC data were treated, if it was necessary, by a deconvolution algorithm to produce separated Gauss shape curves related to the different thermic events using the software of the DSC apparatus.

TGA measurements were performed using an analytical thermobalance PerkinElmer TGA 4000 (Mettler-Toledo, Milano, Italy), operating at a heating rate of 10 °C/min in air atmosphere.

X-ray powder diffraction data were collected with an automatic Philips diffractometer (Malvern Panalytical S.r.l., Lissone (MI), Italy), equipped with a variable temperature camera Anton Paar TTK450 (Rivoli (TO), Italy) and equipped with a standard sample holder for measurements in reflection geometry (depth 500 μm), using Ni-filtered Cu Kα radiation (λ = 1.5418 Å), at a continuous scanning rate of 0.1°(Δ2θ)/14.3s(Δt) with a 2θ diffraction angle. The samples were heated and cooled stepwise at a rate of 10 °C/min to the appropriate temperature, and diffraction data were recorded immediately after reaching the selected temperature.

### 2.3. Photophysical Measurements

Spectrofluorimetric grade solvents were used for photophysical investigations in solution at room temperature. A Perkin Elmer Lambda 900 spectrophotometer (PerkinElmer Inc., Waltham, MA, USA) was employed to obtain the absorption spectra. Steady-state emission spectra were recorded on a HORIBA Jobin-Yvon Fluorolog-3 FL3-211 spectrometer (Horiba Jobin Yvon, Orbassano, Italy) equipped with a 450-W xenon arc lamp, double-grating excitation and single-grating emission monochromators (2.1 nm mm-1 dispersion; 1200 grooves mm^−1^), and a Hamamatsu R928 photomultiplier tube. The glass slab sandwich, containing the LC film sample, was placed into the fluorimeter sample holder on a customized temperature-controlled hot stage realized in Teflon by CaLCTec s.r.l. (Rende, Italy), with 1 °C uncertainty in the temperature. Measurements were performed in a front-face geometry.

Time-resolved measurements were performed using the time-correlated single-photon-counting option on the Fluorolog 3. Laser Nanoled at 379 nm, full-width-half-maximum 750 ps, with repetition rate at 1 MHz, was used to excite the sample. Excitation sources were mounted directly on the sample chamber at 90° to a single-grating emission monochromator (2.1 nm mm^−1^ dispersion; 1200 grooves mm^−1^) and collected with a TBX-04-D single-photon-counting detector. The photons collected at the detector are correlated by a time-to-amplitude converter to the excitation pulse. Signals were collected using an IBH Data Station Hub photon-counting module, and data analysis was performed using the commercially available DAS6 software (HORIBA Jobin-Yvon IBH). Goodness of fit was assessed by minimizing the reduced χ2-function and visual inspection of the weighted residuals.

The emission quantum yields of the samples were obtained by means of a Labsphere optical Spectralon integrating sphere (diameter of 102 mm), which provides a reflectance >99% over 400 to 1500 nm range (>95% within 250–2500 nm). The sphere accessories were made from Teflon (rod and sample holders) or Spectralon (baffle). The sphere was mounted in the optical path of the spectrofluorometer using, as excitation source, a 450 W xenon lamp coupled with a double-grating monochromator for selecting wavelengths. Cylindrical tubes containing the solution samples were placed into the sphere, while the glass slab sandwich containing the LC film sample was placed into the sphere on a customized temperature-controlled hot stage realized in Teflon by CaLCTec s.r.l. (Rende, Italy), with 1 °C uncertainty in the temperature. The procedure used to determine the emission quantum yield was based on the de Mello method [42], slightly modified [43,44]. The emission quantum yield determination is given by:

$$\Phi_{L}=\frac{\left(E_{c\;\;}-\left(1-A\right)E_{a}\right)}{L_{a}\;A}$$

where *A* is the value of the absorbance of the sample at the excitation wavelength measured as:

$$A=\frac{\left(L_{a\;}-L_{c}\right)}{L_{a}}$$

where *L_a_* is the integrated excitation profile when the reference sample is diffusely illuminated by the integrated sphere’s surface; *L_c_* is the integrated excitation profile when the sample is diffusely illuminated by the integrated sphere’s surface; *E_c_* and *E_a_* are the integrated luminescence (corrected for the detector wavelength response) of the sample and the reference sample, respectively, caused by indirect illumination from the sphere. The reference sample is an empty glass slab sandwich. The experimental uncertainties were 1 nm for the band maxima of the luminescence spectra and 5% for the emission quantum yield values.

### 2.4. Molecular Mechanics Calculation

Low-energy model structures of the dimeric building units were obtained using the Biovia Materials Studio program (Milano, Italy) through the utility Forcite. The forcefield COMPASS was used for geometry optimization [45].

## 3. Results

### 3.1. Thermotropic Behavior

As summarized in Table 1, variation in either the anion or the length of the promesogenic chains allowed us to afford a wide mesophases range that spans smectic (SmC and SmA), columnar rectangular (Colr), lamello-columnar (Coll), and nematic discotic/columnar (N_D_/N_C_) ones [29,32,33].

### 3.2. Bulk Photophysics and Thermofluorochromism

**Figure 2:**
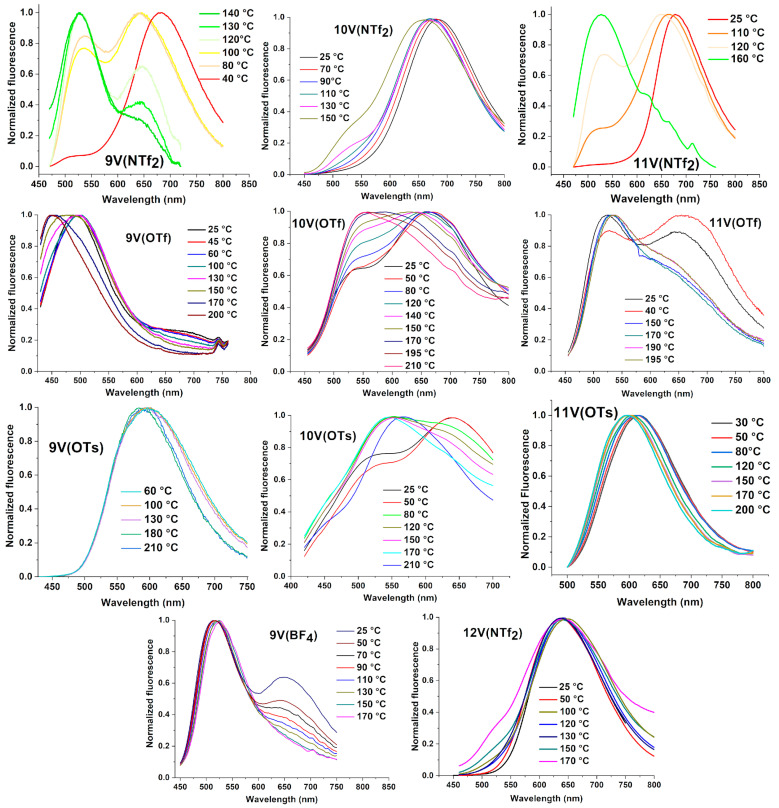
Normalized emission spectra of some thienoviologens recorded at different temperatures in the whole thermotropic range.

All compounds have been characterized in bulk samples from room temperature to the higher transition temperature, and the emission spectra are reported in Figure 2. All of the compounds exhibit significant changes in the fluorescent emission in the LC state by varying the temperature. At room temperature, two emission maxima characterize the spectra of the compounds, one centered in the 620 to 690 nm range (red band) and another peaking in the 485 to 530 nm range (green band). The mutual intensity of the two peaks, as well as the position of the maximum, varies greatly from one sample to another; in some spectra, one of the two peaks appears as a shoulder, and the spectrum seems to consist of a single band (see, for example, the spectra of 9V(OTs), 11V(OTs), 9V(NTf_2_), 10V(NTf_2_), 12V(NTf_2_)). The room temperature emission intensity decay (Table 2) was measured on the most intense peak and, where evident, also on the secondary peak. The best fit was obtained with a three-exponential function for all the triflate and tosylate series, except in the case of 11V(OTs), which is the only one, among the compounds, showing a single emission band (Figure 2), while a double exponential fitting is needed for the bistriflimide series and for 9V(BF_4_). The three lifetime values, ranging between 0.3 and 7.0 ns, imply an overlapping of the red and green bands (Table 2).

The highest emission wavelength has the longest decay time, except for that of 9V(OTf), 10V(OTs), and 11V(OTf).

The emission quantum yields (Table 2) are rather low but substantially similar for all compounds, except for those with NTf_2_^-^ as counterion, which show exceptionally high values, partially tuned by the aliphatic chain length. This behavior has a counterpart in solution, where a concentration-dependent photoluminescence behavior occurs. In dilute solution (CH_2_Cl_2_, 10^−7^ to 10^−5^ mol/L), the thienoviologens show a very intense green fluorescence band (λ_em_~530 nm; λ_ex_ = 450 nm; Φ ≥ 80%; τ = 1–2 ns) (Figure S5 and Table S1), while, for concentrations >10^−5^ mol/L, excitation at 540 nm results in a red emission in the range 640 to 710 nm with a lower quantum yield (up to 3.4%) and a shorter decay lifetime (Table S1), originated by the aggregate form. It is clear that we have more than one emissive species, whose contribution depends on the aggregation/self-assembling properties of the thienoviologen compounds. However, while fluorescence is almost independent on the chain length and of the anion nature in solution, these two structural parameters play a crucial role in the bulk. Indeed, the aggregate formation depends on the distance between two chromophoric units, which is tuned by (i) the volume of the counterion (by increasing counterion volume, the distance increases; see the NTf_2_ case), and (ii) the discoid volume, which, in turn, depends on the mesophase. Notably, these structural properties can be well tuned by the temperature.

At higher temperatures, a relative increase of the green emission at the expense of the red emission and/or a blue shift of the emission band, is generally observed in the bulk fluorescence spectra (Figure 2).

Notably, in most instances, the phase transition can be clearly detected based on the significant spectral changes occurring at the transition point, leading to the formation of a quite definite isosbestic point in the normalized fluorescence spectra (Table 1 and Figure 2 and Figure S1). The emission quantum yield values decrease by increasing temperature, generally halving at the highest temperature value compared to the value measured at room temperature (see Supporting Information).

**Figure 3:**
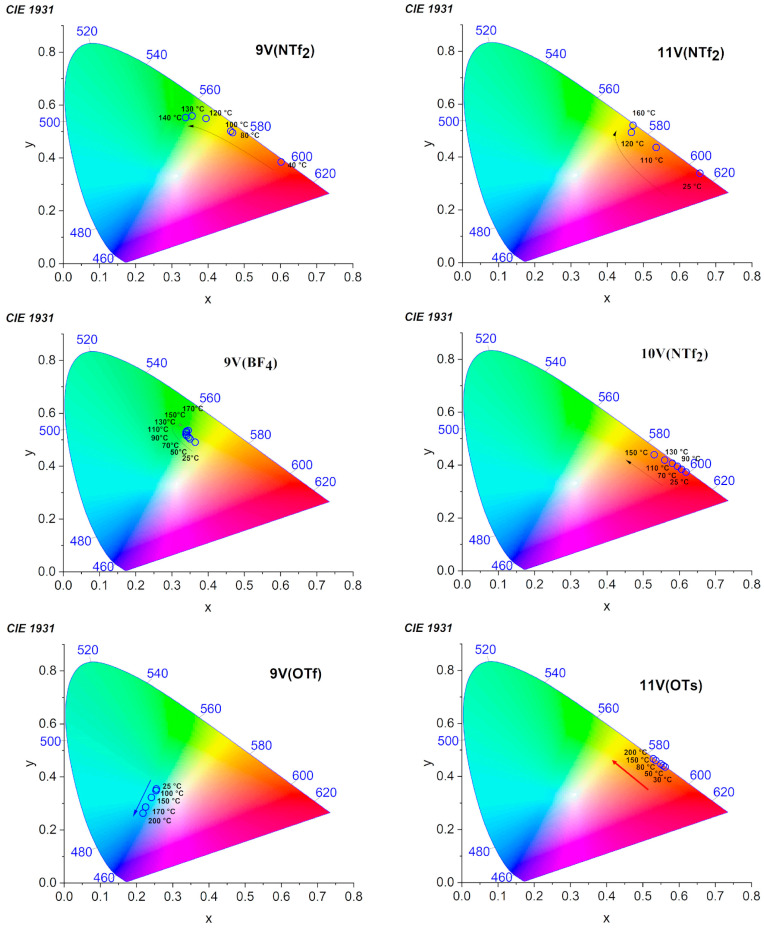
Temperature dependent 1931 CIE chromaticity coordinates of the fluorescent emission of 9V(NTf_2_), 9V(BF_4_), 9V(OTf), 10V(NTf_2_), 11V(NTf_2_), 11V(OTs).

The thermofluorochromic behavior of the mVX is highlighted in the CIE 1931 color space (Figure 3 and Figure S6) and in Table 3. Notably, all of the luminescent ILCs studied show reversible trajectories within the CIE diagram as a function of the temperature, denoting a fine tuning of the emission color in a wide temperature range, made possible by the gradual spectral changes induced by the temperature (Figure 2). Table 3 reports the characteristic color of emission for each phase characterizing the thermotropic behavior of the mVX compounds, thus highlighting the temperature-induced color switching that occurs across each phase transition. Importantly, a temperature-induced switch of the color can be achieved in the mVOTf compounds from green to blue, from orange to yellow, and from yellow to green, as m increases from 9 to 11, from yellow to red in 11VNTf_2_, and, notably, from green to yellow and then to red in the 9VNTf_2_ (Figure 3).

## 4. Discussion

This thermofluorochromic response accounts for the dependence of the emission color on the self-assembling in the LC phase and can be associated with changes in the structural parameters of the liquid crystalline state occurring across the phase transition temperature, even though the color change is almost gradual within the entire thermotropic range from the isotropic liquid state to the crystalline one. By considering the results in solutions, it is clear that the chromophoric unit (i.e., the thienoviologen) shows a green fluorescence (520 nm) with high EQY, which declines with increasing concentration, probably due to the formation of π−π aggregates that quench the emission. On the other hand, in concentrated solution, the thienoviologens show a red emission, originating from the aggregate form. The same behavior is observed in bulk, but in this case the aggregate formation depends on the interaction between two chromophoric units, which is tuned by the volume of the counterion and partially by the chain length. Indeed, two thienoviologen dications have a strong tendency to form disk-like dimers where the counterions are the bridging units through ionic interactions [29]. These dimers are the discoid nanoparticles (approximate dimensions 300 Å2) which constitutes the building blocks for the columnar phases. Aggregate formation is therefore also dependent on the discoid volume, which is modulated by the temperature that determines the type of phase. The formation of higher-order aggregates among the dimers in the bulk LC phase can lead either to a red shift or a blue shift of the light emission, depending on the nature of the intermolecular interactions between the thienoviologens in the dimer, as well as among the dimers in the self-assembled aggregate (cluster) [46].

A classification of the thienoviologens mVX based on their photophysical properties, according to the aliphatic chain length and the nature of the counterion, is not straightforward, although the presence of NTf_2_ implies emission quantum yield (EQY) values that are about two orders of magnitude higher than those of the other counterions (in bulk samples). Actually, the above parameters, in addition to the temperature, combine with differentiated and unpredictable weights to provide the observed photophysical data. However, some general quantitative considerations can be provided in order to try to better understand the thermofluorochromic behavior of these compounds. In general, we observe that, for each compound, a red shift of the fluorescent emission occurs on cooling from the isotropic liquid state, with profound spectral changes and clear isosbestic points in correspondence with the phase transition temperatures, determined by the intensity decrease of the low-energy emission band (650 nm) and the intensity increase of the high-energy one (530 nm).

Moreover, in the triflate and tosylate series, on cooling, the disordered-to-ordered mesophase transition is most often characterized by a change in the 2D space group symmetry from p2mm to p2mg, corresponding to a significant increase in the lattice constant parameters and in the number of salt units from 2 to 4. This corresponds to a quite general increase of the area of the discoid nanoparticles (disk basal area) and a decrease in the ar/br ratio (Table 4). In contrast, in the cases in which the above symmetry transition does not occur (for 9VTf, 10VOTs), a significant decrease in the discoid area can be calculated, which is determined by the decrease of the unit cell parameters a_r_, causing the thermofluorochromic behavior. In all cases, the intracolumnar distance (D) is almost unaffected by any phase transition or change in structural parameters/symmetry (within the second decimal unit) (Table 4). Interestingly, by comparing the columnar ILCs, it can be seen that, in the triflate and tosylate compounds, the intracolumnar distance is smaller than in the corresponding bistriflimide thienoviologens [33,41]. Another interesting difference is in the unit cell parameters, which are larger in the bistriflimide homologous compounds (Table 4).

This may be due to the larger dimension of the NTf_2_^-^ anion, which induces an increase in the intracolumnar distance and in the disk dimensions. This can occur only if the two thienoviologen dication units forming the disk are arranged into larger aggregates with larger intercationic distance and a staggered configuration. This configuration and the larger D values in the mVNTf_2_ series may explain their higher fluorescence quantum yields than the homologous series with the other anions that show significantly lower quantum yields (Table 2).

Summarizing the most important experimental observations, we have: (i) a significant quenching effect of the fluorescence in the solid state (mesophases and crystalline phase) and in concentrated solution for all of the mVX except for the bistriflimide ones; (ii) the appearance of a further band at 540 nm in the absorption spectra, clearly due to aggregation in solution; (iii) a general increase in the contribution of the green emission (blue shift) to the fluorescence spectra at increasing temperature; (iv) a general change (most often a decrease) in the average disk basal area in the columnar compounds with increasing temperature; and (v) the temperature independence of the intracolumnar distance in columnar compounds. All of the above observations could find a qualitative explanation from the knowledge that, when two conjugated chains interact in cofacial configurations (simulating the aggregation in solid state or in concentrated solution), leading to the formation of dimers, fluorescence quenching occurs (aggregation caused quenching) [29,47,48,49,50,51], generally with a resulting blue shift of the lowest optically allowed transition in the dimer with respect to the isolated molecule. This is due to the energy splitting of the excited state energy of the dimer as a consequence of the interaction between the optically allowed energy states of each fluorophore. The lowest excited energy level of the dimer corresponds to a favorable dipole–dipole interaction with an antiparallel arrangement, while, in the highest energy state, corresponds to a parallel orientation of the dipoles. In a cofacial configuration, the transition to the lowest excited state is forbidden, resulting in quenching and a blue shift of the emission with respect to the individual fluorophore. Interestingly, the optical coupling between the ground and excited states in a dimer can be increased in a staggered geometry, wherein the fluorophores in the dimer are translated with respect to one another by a certain amount. In such a case, the lowest excited state corresponds to the allowed optical transition, producing a red shift of the fluorescence compared to the individual fluorophore. Based on this, the geometrical configuration of the fluorophores in the dimer can be assumed to strongly affect the photophysics in the aggregated state. This configuration is affected by the structural parameters *m* and X and mainly by the temperature, which can induce phase transitions leading to important changes in the cofacial configuration of the dimer. Indeed, any increases/decreases in the basal disk area, such as those reported in Table 4, can be accounted for by changes in the geometrical configuration of the dimers in the different mesophases and with a decrease of the disk area attributable to a translation of one thienoviologen with respect to the other from a staggered configuration to a cofacial one upon cooling. Interestingly, this would correspond to a red shift of the fluorescence [29], as we do indeed observe on cooling.

Finally, the strong aggregation-caused quenching effect observed in thienoviologens tosilates, triflates, and tetrafluoroborates does not occur in the blistriflimide homologous series. This can be attributed to the larger van der Waals volume of the latter anion, which acts as bulky units to separate the interacting thienoviologens in the dimer [29,47,48,49,50,51].

**Figure 4:**
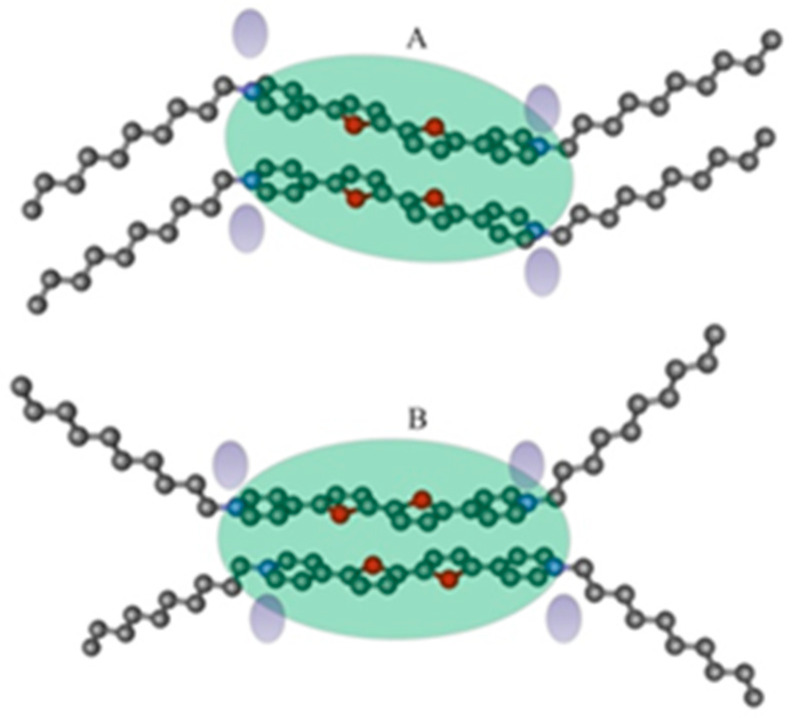
Low-energy models of dimer formed by thienoviologen salt units giving rise to a disc-like motif of elliptic shape (discoid). (**A**): face-to-face configuration where the planar cores have a parallel arrangement of the dipole moments associated with the thiophene rings. (**B**): translation of the planar cores parallel to the long axis and an anti-parallel arrangement of dipole moments associated with the thiophene rings. The counterions are indicated by the shadowed small ovals. The pendant decyl chains are drawn as pointing in the same direction in the zig-zag model A and in opposite directions in B, as an example. In general, they are in a disordered conformation. The intercore distance is 0.4 nm in A and 0.37 nm in B.

Molecular mechanics estimations indicate that the thienoviologens can assume different low-energy cofacial configurations in the dimer, such as those reported in Figure 4, pointing toward a qualitative rationalization of the observed photophysical properties. In model A (Figure 4), the planar cores have a parallel arrangement of the dipole moments associated with the thiophene rings, while model B is characterized by a translation of the planar cores parallel to the long axis and by an anti-parallel arrangement of the dipole moments associated with the thiophene rings. Translation in B allows for alleviating the electrostatic interactions between the positive charges located on the N atom of the pyridyl units, thus originating a blue-shifted emission with respect to A. Additional cofacial arrangements may be easily built with the assumption of an antiparallel arrangement of dipole moments of the thiophene rings for the model A and a parallel arrangement of dipole moments of the thiophene rings for the model B.

## 5. Conclusions

The thienoviologens mVX exhibit a very rich thermotropism, giving rise to a wide range of mesophases, characterized by a dimeric self-assembling unit consisting of two dications and the bridging interacting anions. They exhibit strong fluorescence in dilute solution (up to 90%) but are generally quenched in concentrated solution and in the solid/liquid crystalline state in all the compounds except the bistriflimide ones. In the latter cases, the quenching effect caused by the cofacial configuration of the thienoviologens in the dimer is mitigated by the large anion dimensions. The bulk fluorescence properties of the thienoviologen liquid crystals are determined by the nature of the anion (X), the length of the alkyl chain (m), and the temperature (T). The first two parameters mainly affect the fluorescence properties by determining the configuration in which the dications interact with each other in the aggregates and take into account the different mesomorphism of these compounds across the homologous series. By fixing these two parameters, the effect of temperature changes from room temperature to the isotropic liquid state is revealed by the significant changes of the emission spectra of these compounds, in terms of a modulation of the contribution of the two bands in the green and red regions, characterizing the spectra of all the compounds. Actually, the temperature systematically changes the structural parameters of the phases by changing the geometrical configuration of the fluorophores in the thienoviologen dimer, which is the self-assembling unit in the aggregated state. This leads to a fine tuning of the emission across wide chromaticity coordinates, which can involve the whole visible spectral range.

The results achieved with this work are of noteworthy importance from an application point of view. Indeed, the thermofluorochromic effect is added to the well-known properties of electrochromism and electrofluorochromism in thienoviologens, paving the way for these materials to be excellent candidates in the development of multilevel anti-counterfeiting devices.

In the near future, we will explore different π-conjugated cores and different chains with the main objectives of improving the quantum yield and exploring different termofluorochromic ranges by taking advantage of the high structural versatility of extended viologens.

## Figures and Tables

**Table 1:** Transition temperature and transition enthalpy values (in brackets) for the thienoviologen salts mVX^1^.

Compounds mVX	Transition	Temperature (°C)	[Enthalpy] (J/g)
9V(OTf) ^2^	IL→SmC SmC→Col_L_ Col_L_→K(Col_ro_)	≈180–170 157.5 131.9	[n.d.] ^3^ [13.3] [15.3]
10V(OTf) ^2^	IL→Col_ro1_ Col_ro1_→K(Col_ro2_)	170.1 147.0, 139.1 (double peak)	[13.1] [12.9]
11V(OTf) ^2^	IL→N_C_ N_C_→K(Col_L_)	182.9 147.5	[9.3] [8.0]
9V(OTs) ^2^	SmC→Col_L_/Col_rd_ Col_L_/Col_rd_→K(Col_ro2_)	222.1, 209.4 194.1	[15.9] [13.0]
10V(OTs) ^2^	N_D_/N_C_→Col_rd2_ Col_rd2_→Col_rd1_ Col_rd1_→K(Col_ro_)	229.7 158.7 113.5	[8.1] [3.8] [5.8]
11V(OTs) ^2^	IL→N_D_/N_C_ N_D_/N_C_→K(N_D_/N_C_)	233.0 194.3	[5.9] [15.2]
9V(BF_4_) ^4^	IL→SmA SmA→Glassy SmA	203.0 144.9	[12.0] [9.9]
9V(NTf_2_) ^5^	IL→Col_ro1_ Col_ro1_→Col_ro2_	128.8 109.2	[13.2] [5.2]
10V(NTf_2_) ^5^	IL→SmA SmA→Col_L_	134.2 115.1	[5.8] [0.9]
11V(NTf_2_) ^5^	IL→SmA SmA→Glassy SmA	146.0 114.3	[5.8] [8.8]
12V(NTf_2_) ^6^	IL→SmA SmA→Glassy SmA Glassy SmA→K	190 123.5 108	[n.d.] [6.7] [6.5]

^1^ Determined by DSC analysis at a scan rate of 10 °C min^−1^ in the 2nd cooling scan; abbreviations: K = solid mesophase; Colro and Colrd = ordered and disordered columnar rectangular phases, respectively; Coll = lamello-columnar phase; SmA and SmC = smectic A and smectic C phase, respectively; ND, NC = discotic nematic and columnar nematic phases, respectively; IL = isotropic liquid state. The type of phases was deduced from X-ray diffraction analysis. ^2^ From Ref. [32]. ^3^ Transition not detectable in the DSC thermogram, n.d. = not determined and determined by POM. ^4^ See Figures S1–S4. ^5^ From Ref. [32] and ^6^ from Ref. [33].

**Table 2:** Photophysical properties of the thienoviologens in bulk state at room temperature.

Compound	λ_em_ [nm]	Φ [%]	τ_1_ [ns]	τ _2_ [ns]	τ _3_ [ns]
9V(OTs)	610	0.50	0.70 (10%)	3.40 (76%)	6.90 (14%)
10V(OTs)	535	0.58	0.50 (40%)	2.10 (44%)	5.90 (16%)
	635		0.30 (23%)	1.60 (60%)	4.34 (17%)
11V(OTs)	630	0.54		1.00 (20%)	3.90 (80%)
9V(OTf)	480	0.64	0.26 (87%)	0.90 (10%)	2.50 (3%)
	620		0.45 (45%)	1.49 (50%)	5.33 (5%)
10V(OTf)	530	0.77	0.60 (27%)	1.90 (61%)	6.44 (12%)
	660		0.40 (16%)	1.70 (71%)	5.10 (13%)
11V(OTf)	525	0.84	0.46 (20%)	2.20 (56%)	5.70 (24%)
	640		0.45 (53%)	1.70 (42%)	7.00 (5%)
9V(BF_4_)	520	0.46	0.60 (56%)	2.8 (44%)	
	648		1.00 (43%)	3.1 (57%)	
9V(NTf_2_) ^1^	630	51.0	0.54 (30%)	1.80 (70%)	
10V(NTf_2_)	650	15.0	1.00 (47%)	3.00 (53%)	
11V(NTf_2_) ^1^	670	48.0	0.81 (37%)	2.67 (63%)	
12V(NTf_2_)	647	28.0	1.00 (33%)	3.10 (67%)	

^1^ From Ref. [41].

**Table 3:** Thermofluorochromic behavior of mVX compounds: characteristic colors and CIE coordinates for each phase.

X^-^	m	Phase	CIE Coordinates [T °C]	Coloration
OTf^-^	9	IL	(0.218; 0.263) [200]	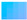
		SmC	(0.241; 0.323) [160]	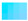
		Col_L_	(0.248; 0.339) [130]	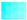
		K(Col_ro_)	(0.254; 0.348) [25]	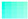
	10	IL	(0.44; 0.489) [195]	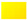
		Col_ro1_	(0.453; 0.481) [150]	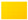
		K(Col_ro2_)	(0.46; 0.462) [25]	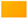
	11	IL	(0.412; 0.506) [190]	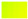
		N_C_	(0.399; 0.509) [150]	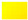
		K(Col_L_)	(0.432; 0.481) [40]	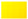
OTs^-^	9	SmC	(0.509; 0.479) [210]	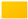
		Col_L_/Col_rd_	(0.513; 0.475) [200]	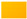
		K(Col_ro2_)	(0.512; 0.475) [100]	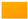
	10	Col_rd2_	(0.399; 0.434) [210]	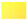
		Col_rd1_	(0.385; 0.420) [120]	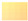
		K(Col_ro_)	(0.413; 0.41) [50]	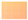
	11	N_D_/N_C_	(0.529; 0.467) [200]	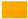
		K(N_D_/N_C_)	(0.561; 0.436) [30]	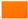
BF_4_^-^	9	SmA	(0.345; 0.535) [170]	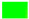
		Glassy SmA	(0.364; 0.491) [25]	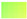
NTf_2_^-^	9	IL	(0.338; 0.553) [140]	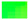
		Col_ro1_	(0.394; 0.549) [120]	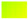
		Col_ro2_	(0.602; 0.385) [40]	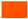
	10	IL	(0.53; 0.439) [150]	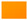
		SmA	(0.559; 0.419) [130]	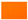
		Col_L_	(0.618; 0.343) [25]	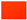
	11	IL	(0.471; 0.52) [160]	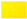
		SmA	(0.535; 0.437) [110]	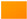
		Glassy SmA	(0.656; 0.339) [25]	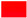
	12	SmA	(0.523; 0.442) [170]	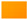
		Glassy SmA	(0.585; 0.407) [120]	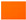
		K	(0.608; 0.389) [25]	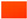

**Table 4:** Effect of temperature on the structural parameters of the mesophases upon the first disordered/ordered phase transition (on cooling from the IL state).

		Space Group Symmetry Changes					
Phases	Compound	Space Group Symmetry ^1^	Number of Salt Units/Cross Section (Z)	Intracolumnar Correlation Distance ^2^ D (Å)	a_r_ (Å)	b_r_ (Å)	Disk Basal Area (Å) ^2^
Columnar	9V(OTf)	p2mg/p2mg	4/4	4.45/4.45	42.40/33.30	30.74/33.34	326/278
	10V(OTf)	p2mm/p2mg	2/4	4.47/4.47	24.07/40.00	26.37/33.34	317/333
	11V(OTf)	p2mm/p2mg	2/4	4.47/4.47	25.14/41.38	27.18/36.06	342/373
	9V(OTs)	p2mm/p2mg	2/4	4.39/4.36	25.79/39.36	25.61/32.13	330/316
	10V(OTs)	p2mg/p2mg	4/4	4.39/4.18	39.36/35.20	32.13/33.34	316/293
	11V(OTs)	p2mm/p2mg/P1	2/4	4.41/4.36	17.85/35.06	36.06/32.65	322/286
	9V(NTf_2_)	p21/a	4/4	4.50	55.8/54.2	22.6/22.0	315/298
	10V(NTf_2_)	SmA/Col_L_	4 (Col_L_)	4.50 (Col_L_)	4.70 (Col_L_)	20.3 (Col_L_)	
Smectic		Interplanar distance (Å)					
	11V(NTf_2_)						
	12V(NTf_2_)			35/34			
	9V(BF_4_)			20.3			

^1^ From Ref [29]; ^2^ From Ref [29] for compounds with X = OTf, OTs and from Ref [32,33] for compound with X = NTf_2_.

## Data Availability

Not applicable.

## Supplementary Materials

The following supporting information can be downloaded at: https://www.mdpi.com/article/10.3390/nano12234284/s1, Scheme S1. Synthesis scheme of the thienoviologens; Figure S1: Representative POM images of the textures of the compound 9VBF_4_ acquired on cooling from the isotropic liquid state at 5 °C/min; Figure S2: DSC thermograms of the sample 9VBF_4_. The phase transitions experienced by the sample in the first heating scan (not reported) are monotropic; Figure S3: TGA of the sample 9VBF4; Figure S4: WAXS profiles of the sample 9VBF_4_, recorded during the second and successive heatings and coolings steps. The phase transitions experienced by the sample in the first heating scan (not reported) are monotropic. The diffraction peak at 2θ ≈ 5° indicates that the smectic period corresponds to 20.3 Å.; Figure S5: Emission spectra in dichloromethane solution at different concentrations; Table S1: Photophysical properties of mVX2 thienoviologens in dichloromethane solution. Figure S6: Temperature-dependent 1931 CIE chromaticity coordinates of the fluorescent emission of 9V(OTs), 10V(OTs), 10V(OTf), 11V(OTf), 12V(NTf_2_).
