# Supplementary material for: The Rainbow Arching over the Fluorescent Thienoviologen Mesophases
Source: Nanomaterials (Basel). 2022 Dec 1;12(23):4284. doi: 10.3390/nano12234284 (PMC9736400; doi:10.3390/nano12234284)
Supplement: Supplementary file 1 [file nanomaterials-12-04284-s001.zip › nanomaterials-2061268-supplementary.pdf]

Supporting Information

# The Rainbow Arching over the Fluorescent Thienoviologen Mesophases

**Giuseppina Anna Corrente [1], Giuseppe Di Maio [2], Massimo La Deda [2,3,*], Odda Ruiz de Ballesteros [4], Bartolo Gabriele [5], Lucia Veltri [5], Finizia Auriemma [4] and Amerigo Beneduci [1,*]**

[1] Laboratory of Physical Chemistry, Materials and Processes for Industry, Environment and Cultural
Heritage, Department of Chemistry and Chemical Technologies, University of Calabria, Via P. Bucci,
Cubo 15D, 87036 Arcavacata di Rende, CS, Italy; giuseppina.corrente@unical.it

[2] Laboratory of Inorganic Molecular Materials, Department of Chemistry and Chemical Technologies,
University of Calabria, Via P. Bucci, Cubo 14C, 87036 Arcavacata di Rende, CS, Italy; giuseppe.dimaio@unical.it

[3] CNR Nanotec, Institute of Nanotechnology, U.O.S. Cosenza, 87036 Arcavacata di Rende, CS, Italy

[4] Dipartimento di Scienze Chimiche, Università di Napoli Federico II, Complesso Monte Sant'Angelo, via Cintia, 80126 Napoli, Italy; ruizdeba@unina.it (O.R.d.B.); auriemma@unina.it (F.A.)

[5] Laboratory of Industrial and Synthetic Organic Chemistry (LISOC), Department of Chemistry and Chemical Technologies, University of Calabria, Via Pietro Bucci 12/C, 87036 Arcavacata di Rende, CS, Italy;
bartolo.gabriele@unical.it (B.G.); lucia.veltri@unical.it (L.V.)

* Correspondence: massimo.ladeda@unical.it (M.L.D.); amerigo.beneduci@unical.it (A.B.)

## Synthesis of the thienoviologens

Viologens were prepared via diquaternization reaction of 5,5-bis(4-pyridyl)-2,2'-bithiophene with iodoalkanes of different alkyl chain length, followed by a metathesis reaction for counterion exchange, as shown in Scheme 1.

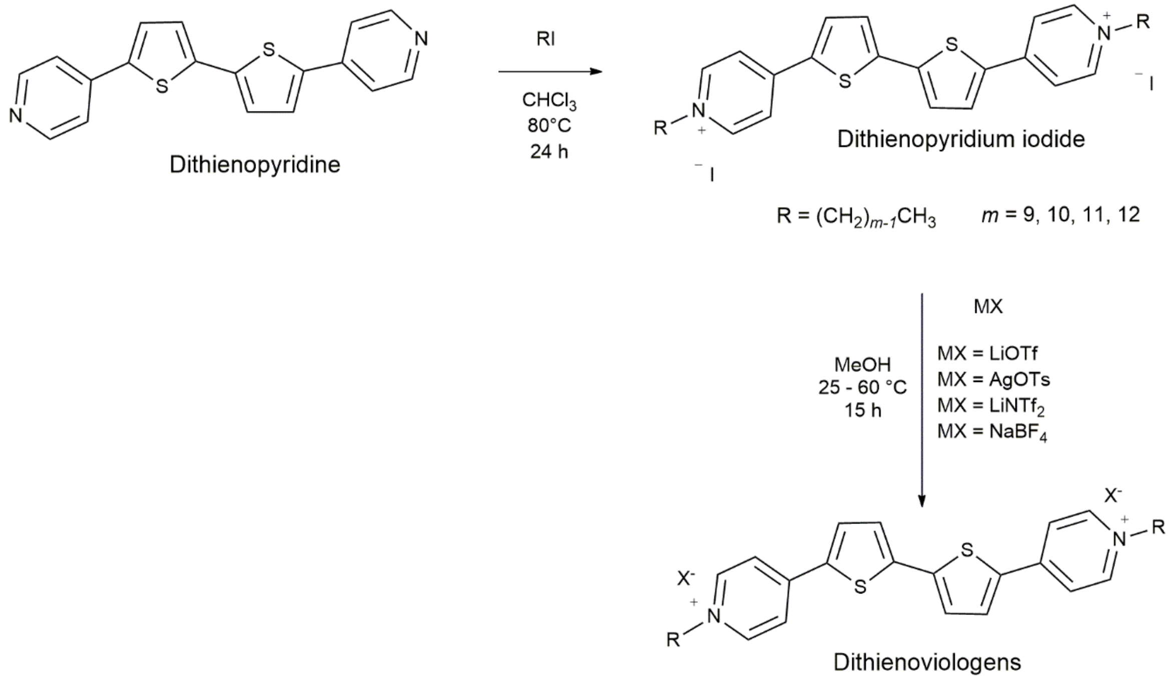


Scheme S1. Synthesis scheme of the thienoviologens

**Synthesis and characterization of 4,4'-(2,2'-bithiophene-5,5'-diyl)bis(1-nonylpyridinium) tetrafluoroborate 9V($BF_4$)**

To a stirred solution of 4,4'-(2,2'-bithiophene-5,5'-diyl)bis(1-nonylpyridinium) iodide (0.2g, 0.24 mmol) in 30 mL of methanol was added sodium tetrafluoroborate (1.6 g, 1.44 mmol). The mixture was stirred at room temperature for 15 h. The solvent was removed under vacuum and to the resulting mixture was added water (50 mL). The suspension was filtered, and the solid residue was washed several times with water (4×10 mL) to give 0.17 g (yield: 93%) of pure thienoviologen tetrafluoroborate **9VBF$_4$**

Orange solid. IR (KBr): ν = 2925 (m), 2858 (m), 1636 (m), 1528 (m), 1435 (m), 1180 (m), 1080 (s), 802 (w) $cm^{-1}$; $^1$H NMR (300 MHz, MeOH-$d_4$): δ = 8.85 (d, $J$ = 7.0, 4 H), 8.30 (d, $J$ = 7.0, 4 H), 8.15 (d, $J$ = 4.1, 2 H), 7.74 (d, $J$ = 4.1, 2 H), 4.54 (t, $J$ = 7.4, 4 H), 2.10-1.95 (m, 4 H), 1.52-1.19 (m, 22 H), 0.90 (t, $J$ = 7.0, 6 H); $^{19}$F NMR (471MHz, MeOH-$d_4$): δ = –152.74, -152.79; HRMS for $C_{36}H_{50}N_2S_2^{2+}$: calc. 574.3404, found 287.1709 (M/2)

**Polarizing optical microscopy and thermal analysis of 9VBF$_4$**

Data on the second thermal cycle. The isotropic liquid state begins at 202 °C and is complete at 226 °C, in agreement to the huge endothermic peak observed in the DSC analysis on the II and III heating cycle at 212 °C (Fig. S2). On cooling from the melt at 5°C/min, bâtonnetes forms at 221 °C (Fig. S1a) and a complete fan shaped texture occurs below 204 °C (Fig. S1b), in agreement with the peak at 203 °C observed in the DSC on the second and third cooling traces (Fig. S2).

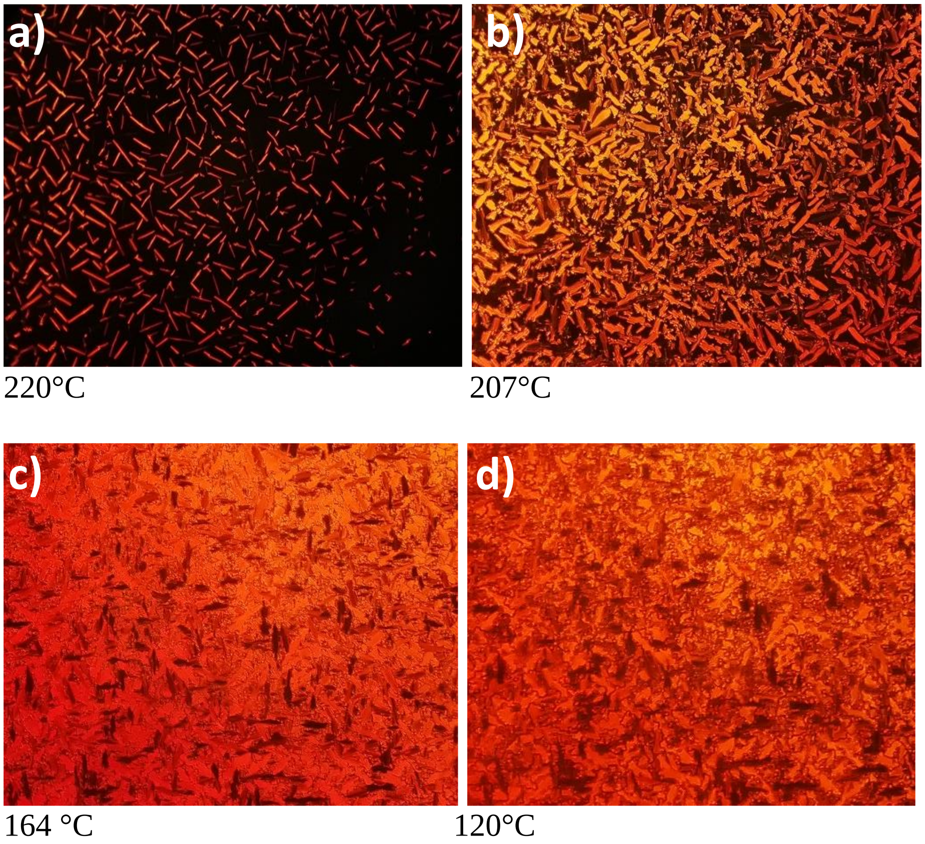


**Figure S1.** Representative POM images of the textures of the compound $9VBF_4$ acquired on cooling from the isotropic liquid state at 5 °C/min.

On further cooling below about 170 °C a poorly developed fan shaped texture is detected, which is almost kept down to room temperature, with only slight changes due to the formation of the the glassy smectic state.

The thermal stability of the sample was studied by thermogravimetric analysis, showed in Figure S3. Thermal decomposition begins at 250 °C ($T_{onset}$) and its 50% decomposition occurs at 382 °C ($T_{d-50\%}$). Sample degradation is complete above 600 °C.

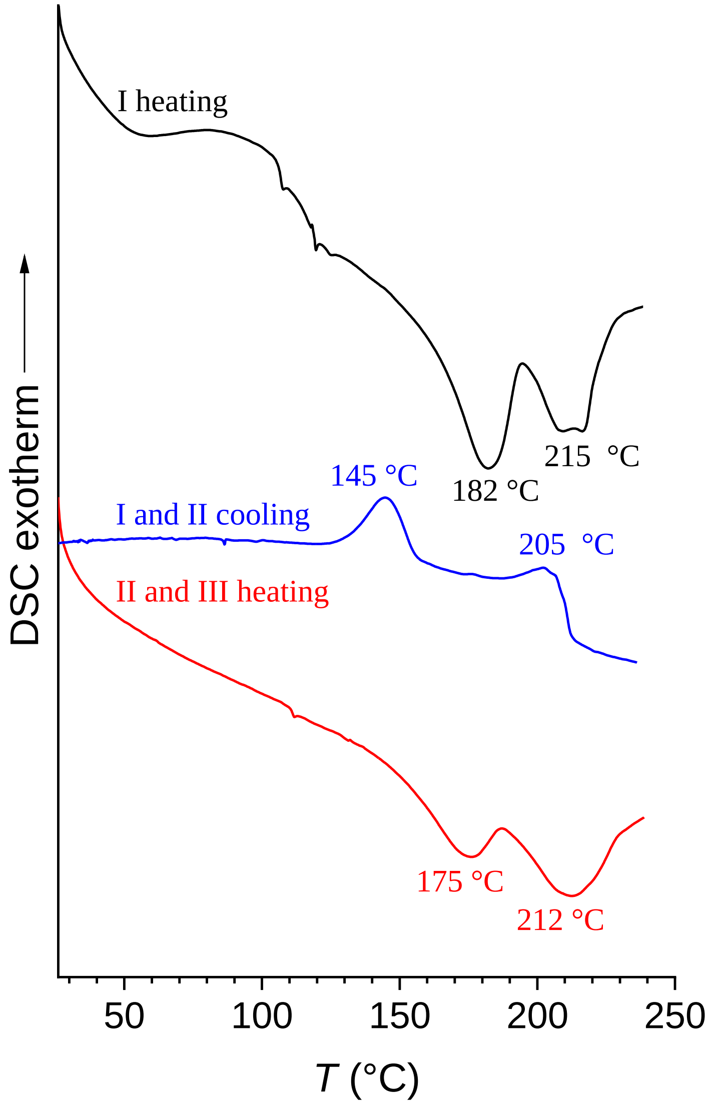
I heating
215 °C
182 °C
145 °C
I and II cooling
205 °C
II and III heating
175 °C
212 °C
DSC exotherm
50
100
150
200
250
T (°C)

**Figure S2.** DSC thermograms of the sample $9VBF_4$. The phase transitions experienced by the sample in the first heating scan (not reported) are monotropic.

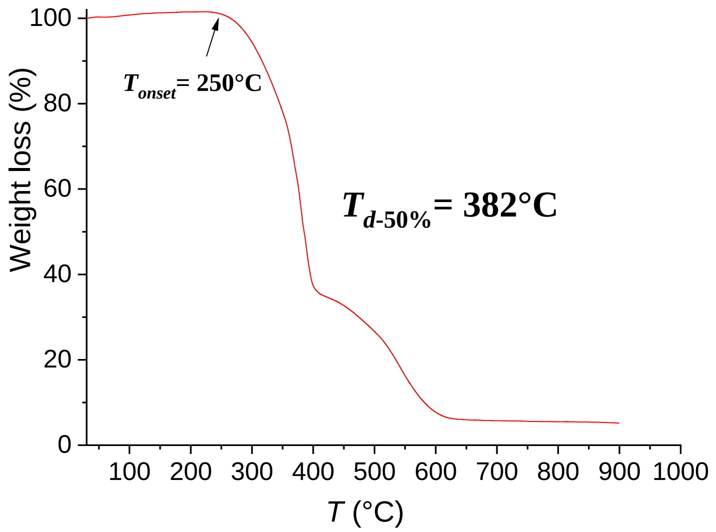


**Figure S3.** TGA of the sample $9VBF_4$.

**XRD analysis**

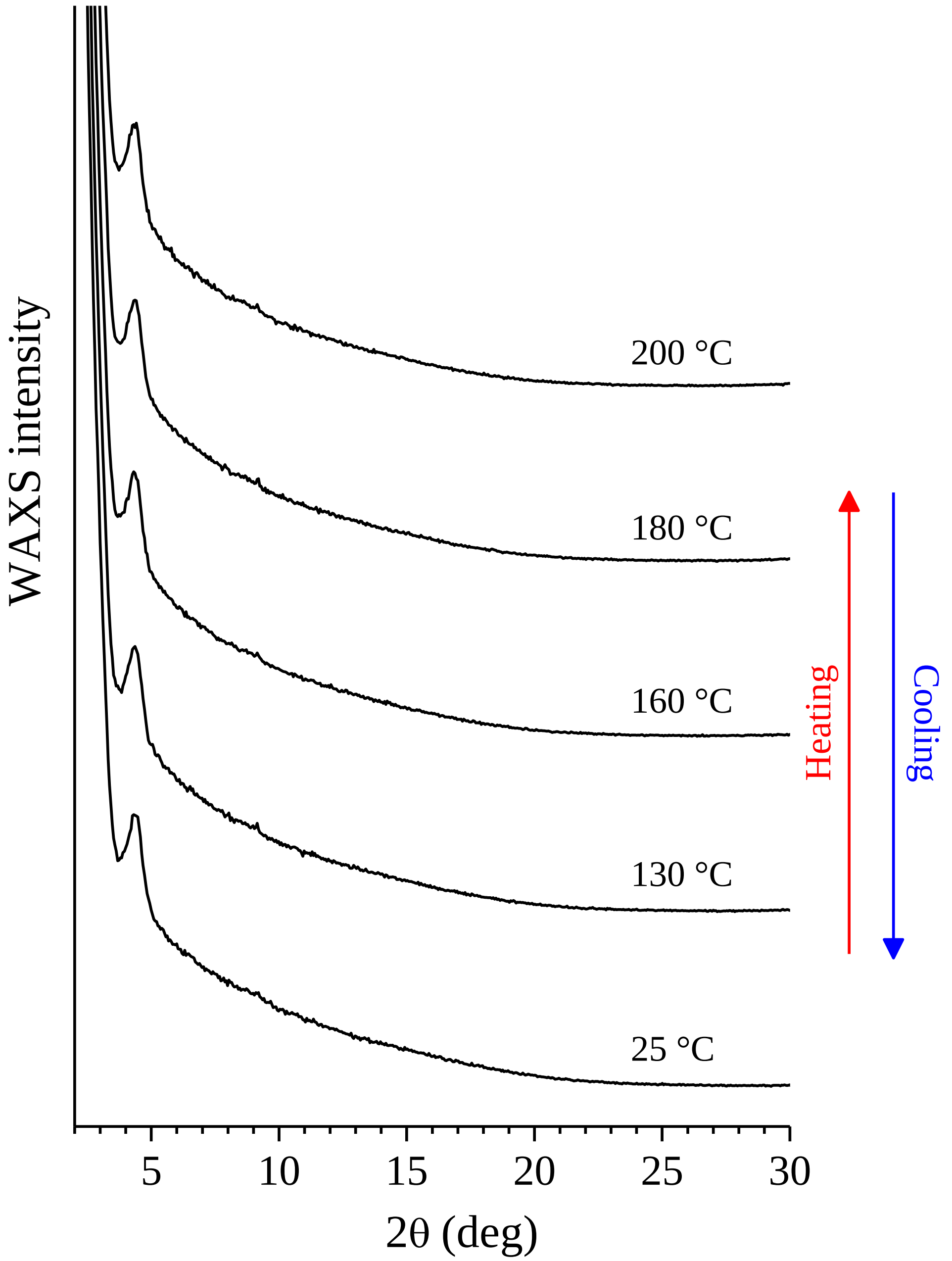


**Figure S4.** WAXS profiles of the sample $9VBF_4$, recorded during the second and successive heatings and coolings steps. The phase transitions experienced by the sample in the first heating

scan (not reported) are monotropic. The diffraction peak at $2\theta \approx 5°$ indicates that the smectic period corresponds to 20.3 Å.

## Photophysical properties in solution

All compounds have been primarily characterized in diluted dichloromethane solution ($10^{-6}$ mol/L). Absorption spectra in the UV-Vis range are almost superimposable, showing a unique band in the range 430-450 nm (which position depends on the counterion or the aliphatic chain length) originated by the $\pi-\pi^*$ transition, typical of charged thienoviologen species (see Tab. S1). [28] The photoluminescence measurements of these compounds in dichloromethane solutions shows a fluorescence band at about 530 nm for concentration ranging from $10^{-7}$ to $10^{-5}$ mol/L. (Fig. S5). The fluorescence quantum yield is very high (ranging from 80 to 99%), with lifetime of about 1 or 2 ns (Table S1).

For concentration $\geq 10^{-5}$ mol/L, the absorption spectra show a characteristic shoulder on the low frequency side at ~ 540 nm (Figure S1). Excitation at 540 nm results in an emission in the range 640-710 nm with a lower quantum yield (up to 3.4%). The decay lifetime is shorter than that measured in diluted solution, with values less than 1 ns (Table S1).

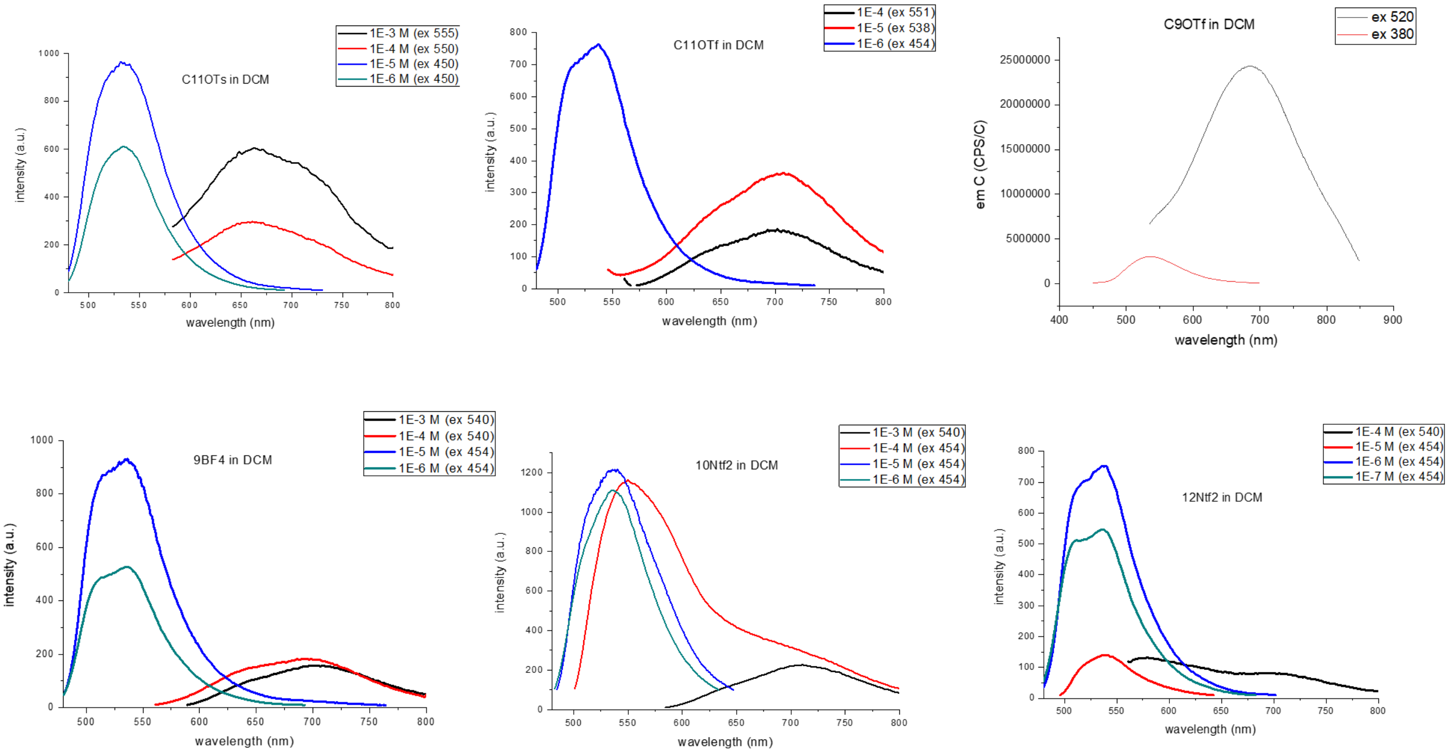

**Figure S5.** Emission spectra in dichloromethane solution at different concentrations.

**Table S1.** Photophysical properties of $mVX_2$ thienoviologens in dichloromethane solution

| | ***Concentration(mol/L)*** | ***Absorption (nm)*** | $\lambda_{ex}$ ***(nm)*** | $\lambda_{em}$ ***(nm)*** | $\Phi$ ***(%)*** | $\tau$ ***(ns)*** |
|---|---|---|---|---|---|---|
| | | | | | | |
| 11V(OTs) | $10^{-6}$ | 436 | 450 | 530 | 87 | 1.21 |
| | $10^{-5}$ | 436 | 450 | 530 | 62 | 1.21 |
| | $10^{-4}$ | 436, 540 | 555 | 700 | 3.38 | < 0.8 |
| | $10^{-3}$ | 436, 540 | 555 | 700 | 0.08 | |
| | | | | | | |
| 11V(OTf) | $10^{-6}$ | 446 | 454 | 534 | 68.7 | 2.35 |
| | $10^{-5}$ | 446 | 538 | 660 | 13.8 | < 0.8 |
| | $10^{-4}$ | 446, 540 | 551 | 660 | 1.23 | < 0.8 |
| | $10^{-3}$ | 446, 540 | | | Too low | |
| | | | | | | |
| 10V(NTf2) | $10^{-6}$ | 450 | 454 | 537 | 98.7 | 2.06 |
| | $10^{-5}$ | 450 | 454 | 537 | 32.9 | 2.06 |
| | $10^{-4}$ | 450, 540 | 540 | 680 | 0.2 | < 0.8 |
| | $10^{-3}$ | 450, 540 | 540 | | Too low | |
| | | | | | | |
| 9V(BF4) | $10^{-6}$ | 446 | 454 | 528 | 79.1 | 1.99 |
| | $10^{-5}$ | 446 | 454 | 528 | 7.39 | 1.99 |
| | $10^{-4}$ | 446, 540 | 540 | 700 | 2.2 | < 0.8 |
| | $10^{-3}$ | 446, 540 | 540 | 700 | 2.0 | < 0.8 |

**Temperature dependence of the fluorescence spectra *vs.* T**

**9VOTf**

The IL/SmC transition can be clearly detected by the appearance of a shoulder at about 500 nm of the main band centred at 450 nm and the isosbestic point at 475 nm (Fig. 2a). Below 150 °C there is a significant intensity increase of the band at about 500 nm at the expense of the low wavelength band, that signs the SmC/ColL transition. On further cooling below 130 °C a new isosbestic points can be detected (605 nm), originated by the appearance of a band at about 680 nm, indicating the formation of the Colro phase. The plain group symmetry does not change from the p2mg. The columnar correlation distance does not significantly change (4.45 Å), but $a_r$ decreases from 42.40 to 33.30 Å while $b_r$ increases from 30.74 to 33.34 Å.

**10VOTf**

A first isosbestic point at 570 nm indicating the IL/Colro1 occurring below 170 °C due to the increase of the emission at 650 nm and the decrease of that at 540 nm. A second isosbestic point occurs at 640 nm below 150 °C due to the Colro1/ Colro2 transition due to the formation of the band at 670 nm and the decrease of the emission at 550 nm (Fig. 2b). The plain group symmetry changes from p2mm to p2mg. The columnar correlation distance does not change (4.47 Å), but $a_r$ increases from 24.07 to 40.00 Å and $b_r$ from 26.37 to 33.34 Å. The number of salt units/cross section (Z) changes from 2 to 4.

**11VOTf**

The IL/Nc transition is evidenced by the decrease of the shoulder at about 650 nm and a slight blue shift of the spectrum. Below 150 °C the isosbestic point at 605 nm due to the formation of the emission band with maximum at 660 nm and the decrease of the intensity of the band at 550 nm, highlights the transition from the Nc to the Colr phase. The plain group symmetry changes from p2mm to p2mg. The columnar correlation distance does not change (4.47 Å), but $a_r$ increases from 25.14 to 41.38 Å and $b_r$ from 27.18 to 36.06 Å. The number of salt units/cross section (Z) changes from 2 to 4.

**9VOTs**

In this case the transition from the disordered columnar phase to the ordered one can be detected by a significant widening of the spectrum and an isosbestic point at about 590 nm. The plain group

symmetry changes from p2mm to p2mg. The columnar correlation distance does not significantly change (4.39 Å to 4.36), but $a_r$ increases from 25.79 to 39.36 Å (at 200 °C and 34.66 at 25 °C) and $b_r$ from 25.61 to 32.13 Å. The number of salt units/cross section (Z) changes from 2 to 4.

**10VOTs**

The PL spectrum was monitored starting from the Nc phase at 170 °C. There are two well defined isosbestic points at 558 nm and at 605 nm. The first occurs due to the transition from the nematic to the disordered columnar phase and the latter is due to the huge increase of the red emission ($\lambda$max = 640 nm) at the expense of the green emission. The plain group symmetry does not change from p2mg. The columnar correlation distance decreases from 4.39 Å to 4.18 Å, $a_r$ decreases from 39.36 to 35.20 Å and $b_r$ increases from 32.13 to 33.34 Å. The number of salt units/cross section (Z) does not changes (Z = 4).

**11OTS**

The transition from the disordered columnar phase to the ordered one can be detected by a significant blue-shift of the spectrum of about 100 nm. The plain group symmetry changes from p2mm to p2mg/p1. The columnar correlation distance decreases from 4.41 Å to 4.36 Å, $a_r$ increases from 17.85 to 35.06 Å (at 200 °C and to 32.14 at 25 °C) and $b_r$ slightly decreases from 36.06 to 34.65 Å. The number of salt units/cross section (Z) changes from 2 to 4.

**9VBF$_4$**

This is a pure smectic compound just below 200 °C. In the SmA phase just below 200 °C its PL spectrum is characterized by an emission band at 530 nm. At the SmA/glassy transition a shoulder appears in the spectrum whichincreases on cooling and has a maximum at about 650 nm. Moreover, the high energy band undergoes a blue shift on cooling.

**9VNTf$_2$**

In the IL state the PL spectrum shows two intense bands at 525 nm and at 640 nm. Upon decreasing the temperature the band at 525 nm progressively decreases on going through the $Col_{ro1}$ phase

(118 °C) to the Col$_{ro1}$ phase (below 109 °C), until ist almost disappearing at room T. Simultaneously, the emission in the red-NIR range, on cooling, shifts to red from about 630 nm to 690 nm.

**10VNTf$_2$**

In the IL state the PL spectrum shows an intense band at 650 nm and a smaller band (shoulder) between 500-550 nm. In the ColL phase at 130 °C the spectrum is similar but the main band is shifted to red and the shoulder is less intense. The emission at 500 disappears on the formation of the Colrd1 phase (below 115 °) and, on further cooling, the spectrum shifted to red.

**11VNTf$_2$**

In the IL state the PL spectrum shows an intense band at 525 nm with a tail extending toward red. Upon decreasing the temperature the band at 525 nm progressively decreases on going through the SmA phase (120 °C) to the glassy SmA phase, until ist almost disappearing at room T. Simultaneously, a relatively higher emission in the red-NIR range occurs which, on cooling, shifts to red from about 630 nm to 670 nm.

**12VNTf$_2$**

This salt forms a SmA phase from the first cooling scan below 180 °C. In the SmA phase its PL spectrum is characterized by an emission band at 650 nm with a small shoulder at 500-550 nm. On cooling, at the SmA/glassy transition (about 125 °C) the shoulder disappears in the spectrum and no other relevant changes could be observed.

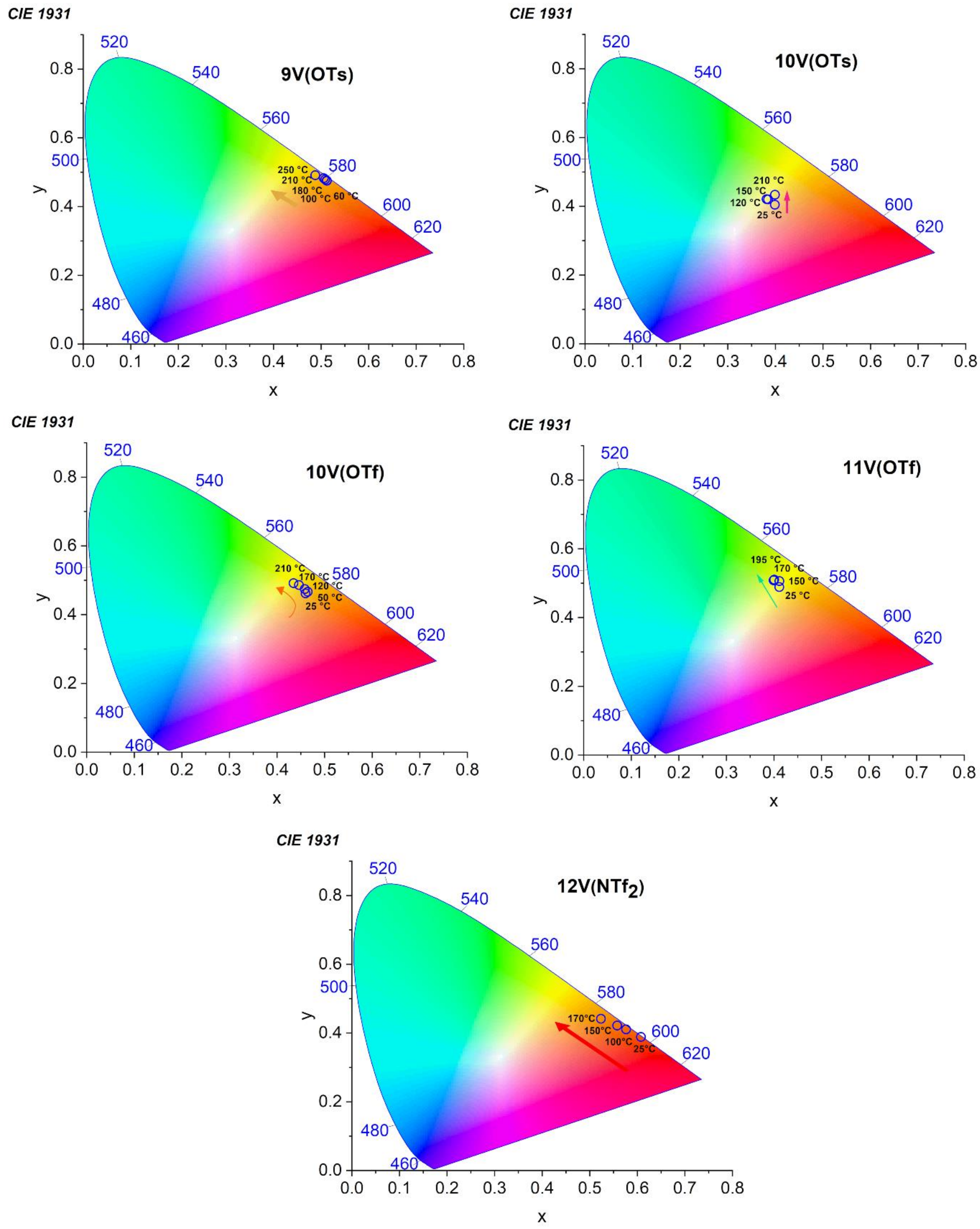


**Figure S6.** Temperature dependent 1931 CIE chromaticity coordinates of the fluorescent emission of 9V(OTs), 10V(OTs), 10V(OTf), 11V(OTf), 12V($NTf_2$).
